# Escore SAGE em Normotensos e Pré-Hipertensos: Uma Prova de Conceito

**DOI:** 10.36660/abc.20220291

**Published:** 2023-02-13

**Authors:** Rayne Ramos Fagundes Rigonatto, Priscila Valverde Oliveira Vitorino, Adriana Camargo Oliveira, Ana Luiza Lima Sousa, Paulo César Brandão Veiga Jardim, Pedro Miguel Guimarães Marques Cunha, Eduardo Costa Duarte Barbosa, Panagiotis Xaplanteris, Charalambos Vlachopoulos, Weimar Kunz Sebba Barroso

**Affiliations:** 1 Universidade Federal de Goiás Programa de Pós-graduação em Ciências da Saúde Goiânia GO Brasil Universidade Federal de Goiás – Programa de Pós-graduação em Ciências da Saúde, Goiânia, GO – Brasil; 2 Pontifícia Universidade Católica de Goiás Escola de Ciências Sociais e da Saúde Goiânia GO Brasil Pontifícia Universidade Católica de Goiás – Escola de Ciências Sociais e da Saúde, Goiânia, GO – Brasil; 3 Universidade Federal de Goiás Liga de Hipertensão Arterial Goiânia GO Brasil Universidade Federal de Goiás – Liga de Hipertensão Arterial, Goiânia, GO – Brasil; 4 Escola de Medicina Universidade do Minho Braga Portugal Escola de Medicina da Universidade do Minho, Braga – Portugal; 5 Complexo Hospitalar Santa Casa de Misericórdia de Porto Alegre Porto Alegre RS Brasil Complexo Hospitalar Santa Casa de Misericórdia de Porto Alegre, Porto Alegre, RS – Brasil; 6 Université Libre de Bruxelles Cardiology Department Bruxelas Bélgica Université Libre de Bruxelles – Cardiology Department, Bruxelas – Bélgica; 7 National and Kapodistrian University of Athens School of Medicine Department of Cardiology Atenas Grécia National and Kapodistrian University of Athens School of Medicine – First University Department of Cardiology, Atenas – Grécia

**Keywords:** Hipertensão, Biomarcadores, Rigidez vascular, Análise de onda de pulso, Fatores de risco

## Abstract

**Fundamento:**

O SAGE foi desenvolvido para identificar hipertensos com chance de velocidade de onda de pulso (VOP) aumentada. Até o momento, as publicações do escore foram em hipertensos.

**Objetivo:**

Verificar a capacidade do SAGE de identificar os normotensos ou pré-hipertensos com chance de aumento da VOP.

**Métodos:**

Transversal retrospectivo, incluiu exames de normotensos e pré-hipertensos que realizaram a medida central da pressão arterial e apresentavam os parâmetros para o cálculo do escore. Para cada pontuação do escore, foi analisada a sensibilidade, especificidade, valor preditivo positivo e negativo utilizando como ponto de corte para o diagnóstico positivo VOP ≥ 10m/s, ≥9,08 m/s (percentil 75) e ≥7,30 m/s (percentil 50). Um valor de p<0,05 foi adotado como estatisticamente significante.

**Resultados:**

A amostra foi de 100 participantes normotensos ou pré-hipertensos, com média (DP) de 52,64 (14,94) anos e VOP mediana de 7,30 m/s (6,03 – 9,08). O SAGE apresentou correlação com idade (r=0,938, p<0,001), glicemia (r=0,366, p<0,001) e taxa de filtração de glomerular (r=-0,658, p<0,001). A área sob a curva ROC foi de 0,968 (p<0,001) para VOP≥10 m/s, 0,977 (p<0,001) para VOP≥9,08 m/s e 0,967 (p<0,001) para VOP≥7,30 m/s. O escore 7 apresentou especificidade de 95,40% e sensibilidade de 100% para VOP≥10 m/s. O ponto de corte seria cinco para VOP≥9,08 m/s (s=96,00%, e= 94,70%), e dois para VOP≥7,30 m/s.

**Conclusão:**

O SAGE foi capaz de identificar indivíduos com maior chance de apresentar rigidez arterial, utilizando diferentes pontos de corte de VOP. Entretanto, o desenvolvimento de um escore específico para normontensos e pré-hipertensos faz-se necessário.

## Introdução

A velocidade de onda de pulso (VOP) é um biomarcador consolidado na estratificação do risco cardiovascular e identificação de lesões subclínicas. Quando maior que 10 m/s, a VOP também significa lesão em órgão alvo.^1 - 4^ Entretanto, ainda é subutilizada na prática clínica pelo alto-custo e pela baixa disponibilidade de equipamentos de avaliação.^5^

O escore SAGE foi desenvolvido com o intuito de difundir o conhecimento e o conceito da avaliação de dano e envelhecimento vascular, utilizando quatro parâmetros simples – idade, pressão arterial sistólica (PAS), glicemia de jejum e taxa de filtração glomerular (TFG) – para calcular a possibilidade de o indivíduo apresentar aumento da rigidez arterial. De acordo com a pontuação obtida, pode-se encaminhar com maior assertividade os pacientes para a realização da medida central da pressão arterial (MCPA) e análise da VOP.^5^

No estudo de desenvolvimento do escore, o ponto de corte do SAGE foi calculado utilizando a VOP carotídeo-femoral na população hipertensa.^5^ Em seguida, o escore foi calculado usando a VOP tornozelo braquial em japoneses hipertensos^6^ e, recentemente, o escore foi calculado utilizando o método oscilométrico, mais difundido no Brasil, com brasileiros hipertensos.^7^

Em 2021, um estudo^8^ com 760 chineses desenvolveu um novo escore clínico utilizando idade, PAS periférica (PASp) e pressão arterial diastólica periférica (PADp), peso e altura, também visando identificar os indivíduos com aumento da rigidez arterial. Porém, esse estudo foi feito especificamente com diabéticos, e utilizou a análise da VOP tornozelo-braquial.

Há ainda uma lacuna na literatura em relação ao uso desses escores para identificar o aumento da rigidez arterial nos indivíduos não hipertensos, mas que já podem apresentar VOP elevada e risco de desfechos cardiovasculares, e, de estudos utilizando o método oscilométrico, que apresenta menor custo e é de fácil aplicação.

Diante do exposto, o objetivo do estudo foi verificar a capacidade do escore SAGE em identificar a chance de aumento da VOP em uma amostra de brasileiros normotensos ou pré-hipertensos como uma prova de conceito.

## Métodos

### Tipo de estudo e local da pesquisa

Estudo transversal que avaliou prontuários de pacientes de dois centros de referência no diagnóstico e acompanhamento de hipertensão arterial no Brasil.

### População e amostra

No período de setembro de 2012 a novembro de 2019, foram realizadas 1594 avaliações da MCPA pelo método oscilométrico. Desses exames foram excluídos:

- os de participantes menores de 18 anos;
- os de hipertensos com diagnóstico de hipertensão arterial definido como PASp ≥ 140 mmHg e/ou PADp ≥ 90 mmHg, ambos obtidos na MCPA; ou PAS média ≥ 130 mmHg e/ou PAD média ≥ 80 mmHg (PAD) na monitorização ambulatorial da pressão arterial (MAPA)^9 , 10^ ou uso de medicações anti-hipertensivas;
- os pacientes que não apresentavam os parâmetros clínicos e laboratoriais necessários para o cálculo do escore SAGE (PASp, idade, glicemia de jejum e creatinina para obtenção do *clearance* de creatinina segundo o grupo CKD-EPI).^5^ Os exames laboratoriais deveriam ter sido realizados até três meses antes ou após a MCPA;
- os de pacientes com *clearance* de creatinina calculado pelo grupo CKD-EPI inferior a 15 ml/min/1,73m^2^.^5^

**Figura 1:**
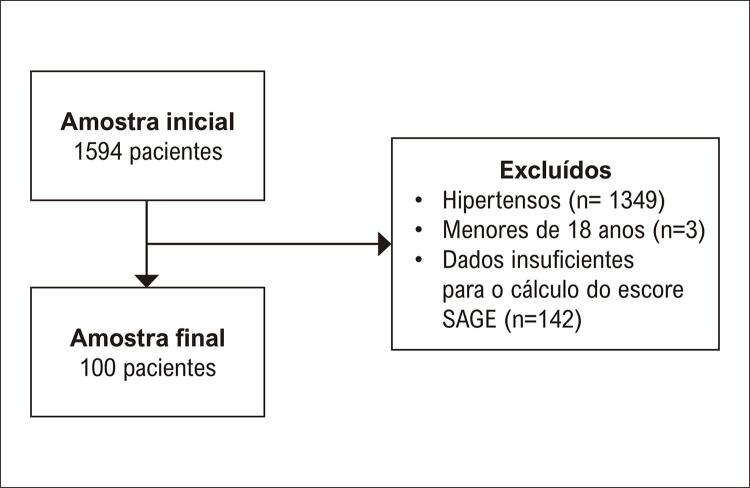
– Fluxograma de seleção dos participantes. Fonte: Autor, 2022.

Portanto, a amostra foi constituída por 100 indivíduos normotensos ou pré-hipertensos, que apresentavam todos os dados necessários para o cálculo do escore SAGE ( Figura 1 ).

### Procedimentos do estudo

Foram identificados, nos dois centros de referência, todos os exames em arquivo eletrônico de MCPA realizados entre setembro de 2012 a novembro de 2019. Em seguida, foram analisados os prontuários dos pacientes para verificar os critérios de elegibilidade do estudo.

Dentre os elegíveis, os seguintes dados foram coletados do exame da MCPA: data de nascimento, data de realização do exame, peso, altura, PAS e PAD periféricas e centrais, pressões de pulso periférica e central, *Augmentation Index* (AIx) e VOP. Para os parâmetros centrais e periféricos, a média de três medidas foi considerada para a análise.

Além disso, foram coletados os seguintes dados: sexo, tabagismo, sedentarismo, estado civil, dados sobre os medicamentos em uso, diagnósticos clínicos, e os resultados dos exames de glicemia jejum e creatinina realizados três meses antes ou após o exame da MCPA. Nos casos em que o mesmo exame foi realizado mais de uma vez nesse período, foi considerado o mais próximo da data de realização da MCPA.

O índice de massa corporal foi calculado utilizando a fórmula: $\text{peso}(Kg)/[\mathrm{altura}(m){]}^{2}$ .^11^

### Avalição da medida central da pressão arterial

A avaliação da MCPA foi realizada com o Mobil-O-Graph^®^ (IEM, Stolber, Alemanha) e com o Dyna MAPA AOP^®^ (Cardios, São Paulo, Brasil). Essa avaliação é feita de forma não invasiva, os parâmetros periféricos (pressão sistólica e diastólica) são aferidos com esfigmomanômetro e o algoritmo ARCSolver é utilizado para estimar o valor da pressão arterial a nível central.^12^

A avaliação da VOP com uso da braçadeira, pelo método oscilométrico, apresenta valores semelhantes aos obtidos de forma invasiva com cateter intra-aórtico,^12^ e é mais reprodutível que os equipamentos de avaliação da VOP carotídeo-femoral.^13^ Ela também é validada para a avaliação da PAS central em comparação com a avaliação pelo método invasivo e pelo método tonométrico.^14^

Aumento da rigidez arterial compatível com lesão em órgão-alvo foi identificada pela VOP maior ou igual a 10 m/s.^9 , 15^

### Cálculo do escore SAGE

**Figura 2:**
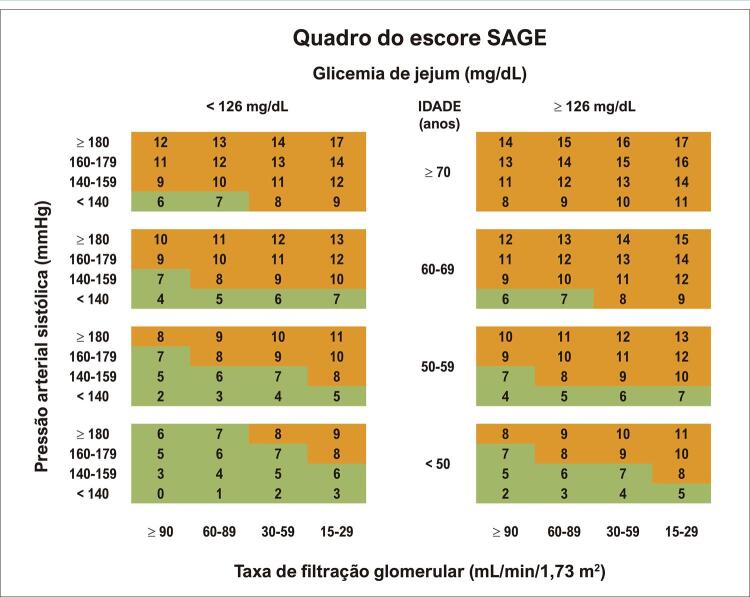
– Identificação do escore SAGE de acordo com as quatro variáveis que o constituem; traduzido de Xaplanteris et al.5

O SAGE escore é definido a partir de quatro variáveis: glicemia de jejum, PASp, idade e taxa estimada de filtrado glomerular ( Figura 2 ).

Por exemplo, um indivíduo com pressão sistólica de 145 mmHg, glicemia de 110 mg/dl, 65 anos e TFG de 69 mL/min/1,73m^2^ receberá o escore SAGE 8 e, portanto, conforme definido pelo estudo de desenvolvimento do escore, será indicado para realizar a avaliação da rigidez arterial, devido à maior chance de seu aumento.^5^

O SAGE foi calculado para cada um dos participantes. A PASp foi obtida no exame de MCPA, a idade foi estimada pela diferença obtida a partir da diferença entre a data de realização desse exame e a data de nascimento dos participantes. A glicemia foi coletada do prontuário e a TFG foi calculada utilizando a fórmula CKD-EPI 2021, a partir dos valores de creatinina coletados do prontuário.

### Análise estatística

Os dados foram coletados por duas pesquisadoras, utilizando um formulário elaborado no programa Epidata, versão 3.1.^16^ Também por meio do programa, a dupla digitação dos dados foi validada para a verificação de possíveis inconsistências e correção.

Os dados foram analisados com o *Statistical Package for Social Science* versão 23.0 (SPSS). Foi aplicado teste de Kolmogorov-Smirnov para verificar a normalidade de distribuição dos dados e foi realizada a análise descritiva dos dados, utilizando média e desvio-padrão para os dados paramétricos e mediana e intervalo interquartil para os não paramétricos. Os dados qualitativos foram apresentados como frequências absoluta e relativa.

A correlação entre o SAGE e cada uma das quatro variáveis que o compõe foi realizada com o teste de correlação de Spearman.

A análise de sensibilidade, especificidade, valor preditivo positivo e negativo foi realizada para cada pontuação SAGE, utilizando como diagnóstico positivo três valores de VOP ≥ 10m/s e VOP ≥ percentis 50 e 75, que corresponderam a 7,3 e 9,8 m/s, respectivamente. Para cada um desses valores foi construída a curva ROC e definido o melhor ponto de corte do escore SAGE, isto é, aquele com maior sensibilidade e especificidade para a identificação dos pacientes com maior chance de VOP elevada. Considerou-se como significativo p<0,05.

### Aspectos éticos

A pesquisa seguiu as normas da resolução nº 466/12 e foi aprovada pelo Comitê de Ética do Hospital das Clínicas da Universidade Federal de Goiás sob os pareceres nº 1.500.463 e 3.792.750 (emenda), com dispensa do Termo de Consentimento Livre e Esclarecido (TCLE).

## Resultados

Foram analisados dados de 100 participantes, com média de idade 52,64 ± 14,94 anos. A maioria dos participantes eram do sexo masculino, com dislipidemia, com PA ótima e valores de VOP inferiores a 8 m/s ( Tabela 1 ).

**Tabela 1:** – Características sociodemográficas e clínicas dos participantes (n=100)

Variável	n/%
**Sexo**	
Feminino	45
Masculino	55
**Estado civil**	
Sem companheiro	29
Com companheiro	57
Não informado	14
**Idade**	
< 50 anos	42
50 a 59 anos	24
60 a 69 anos	18
≥70 anos	16
**Fator de risco**	
Fumante	5 / 5,3%*
IMC > 30Kg/m^2^	24
Diabetes Mellitus	11 / 11,7%*
Dislipidemia	53 / 65,4%*
**Colesterol total**	
< 150 mg/dl	29
150 - 199 mg/dl	41
200 - 249 mg/dl	18
250 - 299 mg/dl	4
≥ 300 mg/dl	1
Não informado	7
**LDL**	
≤50 mg/dl	8
51–69 mg/dl	10
70–99 mg/dl	26
100–129 mg/dl	31
≥130 mg/dl	16
Não informado	9
**Triglicérides**	
<150 mg/dl	63
≥150 mg/dl	26
Não informado	11
**Glicemia**	
< 126 mg/dl	94
≥ 126 mg/ dl	6
**Taxa de Filtração Glomerular**	
30 a 59	5
60 a 89	48
≥ 90	47
**Classificação da Pressão Arterial**	
PA ótima	46
PA normal	34
Pré-hipertensão	20
**Rigidez arterial**	
VOP < 8 m/s	59
VOP 8 - 10 m/s	29
VOP > 10 m/s	12
**Parâmetros de Pressão Central**	**Média (DP) / Mediana (25 – 75)**
PASp (mmHg)	119,43 (9,59)
PADp (mmHg)	75,50 (67,00 – 79,75)
PPp (mmHg)	45,00 (39,00 – 52,00)
PASc (mmHg)	109,15 (9,38)
PADc (mmHg)	77,00 (67,25 – 81,00)
PPc (mmHg)	32,00 (29,00 – 39,00)
AI (%)	18,87 (11,30)
VOP (m/s)	7,30 (6,03 – 9,08)

*Alguns pacientes não continham esses dados, logo a frequência foi diferente do n. AI(%): augmentation index; HDL: lipoproteína de alta densidade; IMC: índice de massa corporal; LDL: lipoproteína de baixa densidade; PADc: pressão arterial diastólica central; PADp: pressão arterial diastólica periférica; PASc: pressão arterial sistólica central; PASp: pressão arterial sistólica periférica; PPc: pressão de pulso central; PPp: pressão de pulso periférica; VOP: velocidade da onda de pulso

O escore SAGE mais frequente na amostra foi 0, seguido de 1 e 2 ( Figura 03 ). Ao verificar as características desses pacientes que justifiquem o escore, verificou-se que dentre os 13, 12 participantes estavam na faixa etária de 70 anos ou mais e apresentavam glicemia < 126mg/dL e TFG de 60 a 89 mL/min/1,73m^2^. O outro paciente apresentou idade entre 60 e 69 anos, glicemia ≥ 126mg/dL e a mesma TFG.

**Figura 3:**
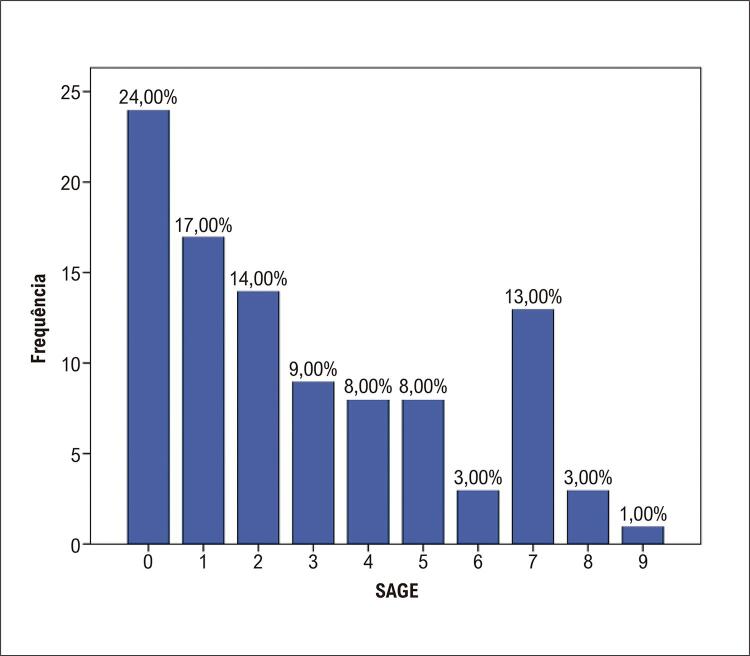
– Frequência relativa de distribuição do escore SAGE (n=100).

**Figura 4:**
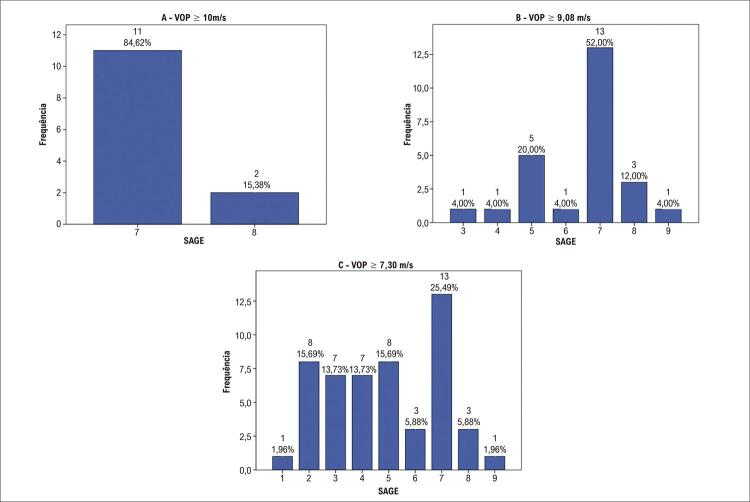
– Frequência absoluta e relativa de distribuição do escore SAGE, dentre os participantes ; (A) pacientes com velocidade de onda de pulso (VOP) ≥ 10 m/s, (B) pacientes com VOP ≥ 9,08 m/s e (C) pacientes com VOP ≥ 7,30 m/s.

Entre os pacientes com rigidez arterial (VOP ≥ 10 m/s), o escore mais frequente foi sete ( Figura 4 ). Todos os pacientes com VOP ≥ 10 m/s (n=13, 100%) apresentavam 70 anos ou mais e glicemia < 126 mg/dL; 10 (76,9%) apresentagvam TFG entre 60 e 89 ml/min/1,73m^2^, e três (23%) entre 30 e 59 ml/min/1,73m^2^. Onze (84,6%) apresentavam dislipidemia.

Analisando-se o percentil 75 (9,08 m/s) e o percentil 50 da VOP (7,30 m/s), o escore SAGE mais frequente também foi sete. Dentre os pacientes com VOP ≥ 9,08 m/s, 88% tinham glicemia < 126 mg/dL, 64% tinha 70 anos ou mais (e os demais estavam na faixa etária entre 60 e 69 anos), e 80% tinham TFG entre 60 e 89 ml/min/1,73m^2^.

**Figura 5:**
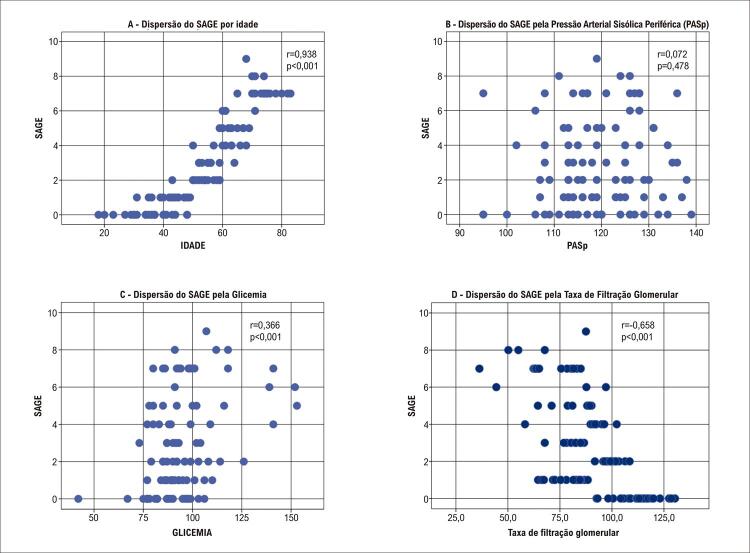
– Distribuição do SAGE (A- de acordo com a idade, B- de acordo com a PASp, C- de acordo com a taxa de glicemia, D- de acordo com o CKD-EPI).

A distribuição dos parâmetros de SAGE de acordo com a idade, a PASp, a glicemia de jejum e a TFG (segundo grupo CKD-EPI) ( Figura 5 ), demonstrou correlação positiva com a idade e com a taxa de glicemia, e correlação negativa com a TFG. Não foi encontrada correlação entre o SAGE e a PASp.

**Figura 6:**
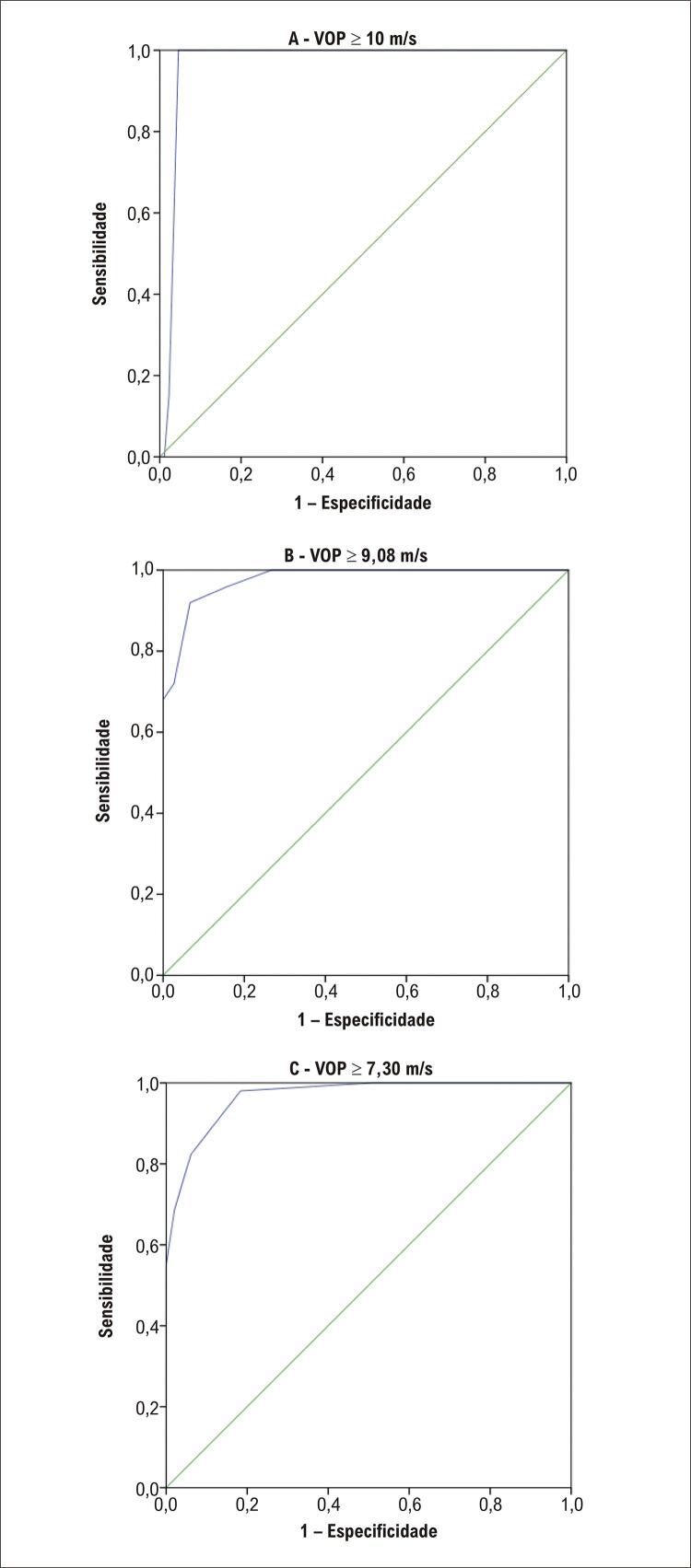
– Curva ROC do escore SAGE (A - para VOP ≥ 10 m/s, B – para VOP ≥ 9,08 m/s e C – para VOP ≥ 7,30 m/s.

Na análise da curva ROC, a área sob a curva para VOP ≥ 10 m/s, foi de 0,968 (p<0,001), para VOP ≥ 9,08 m/s foi 0,977 (p<0,001) e para a VOP ≥ 7,30 m/s foi 0,967 (p<0,001) ( Figura 6 ).

De acordo com a análise de sensibilidade e especificidade ( Tabela 2 ), para os indivíduos com rigidez arterial (VOP ≥ 10 m/s), o escore sete apresentou alta especificidade (95,40%) associado à uma sensibilidade de 100% e um valor preditivo negativo de 100%. Considerando o percentil 75 (VOP ≥ 9,08 m/s), o ponto de corte seria o SAGE ≥ 5, com sensibilidade de 96,00% e especificidade de 94,70%. Já para a mediana da VOP (≥ 7,30 m/s), o ponto de corte reduziria para dois.

**Tabela 2:** – Sensibilidade e especificidade do escore SAGE por ponto de corte e considerando diferentes valores de velocidade da onda de pulso

	SAGE	Sensibilidade	Especificidade	VPP/ Corretamente classificado	VPN
**VOP ≥ 10 m/s**	0	100,00%	0,00%	13,00%	-
	1	100,00%	27,59%	17,11%	100,00%
	2	100,00%	47,13%	22,03%	100,00%
	3	100,00%	63,22%	28,89%	100,00%
	4	100,00%	73,56%	36,11%	100,00%
	5	100,00%	82,76%	46,43%	100,00%
	6	100,00%	91,95%	65,00%	100,00%
	7	100,00%	95,40%	76,47%	100,00%
	8	23,08%	98,85%	75,00%	89,60%
	9	0,00%	98,85%	0,00%	86,90%
**VOP ≥ 9,08 m/s**	0	100,00%	0,00%	25,00%	-
	1	100,00%	32,00%	32,89%	100,00%
	2	100,00%	54,70%	42,40%	100,00%
	3	100,00%	73,30%	55,60%	100,00%
	4	100,00%	85,30%	69,40%	100,00%
	5	96,00%	94,70%	85,70%	98,60%
	6	72,00%	97,30%	90,00%	91,30%
	7	68,00%	100,00%	100,00%	90,40%
	8	16,00%	100,00%	100,00%	78,10%
	9	4,00%	100,00%	100,00%	75,80%
**VOP ≥ 7,30 m/s**	0	100,00%	0,00%	51,00%	-
	1	100,00%	48,98%	67,11%	100,00%
	2	98,00%	81,60%	84,70%	97,60%
	3	82,40%	93,90%	93,30%	83,60%
	4	68,60%	98,00%	97,20%	75,00%
	5	54,90%	100,00%	100,00%	68,10%
	6	39,20%	100,00%	100,00%	61,30%
	7	33,30%	100,00%	100,00%	59,00%
	8	7,80%	100,00%	100,00%	51,00%
	9	2,00%	100,00%	100,00%	49,50%

*VOP: velocidade de onda de pulso; VPN: valor preditivo negativo, VPP: valor preditivo positivo.*

## Discussão

No presente estudo identificamos que o escore 0 foi o mais frequente no total da amostra de não hipertensos. Por outro lado, naqueles com VOP ≥ 7,3 m/s, 9,08 m/s ou 10 m/s o escore sete foi o mais frequente. O escore SAGE apresentou correlação positiva e moderada com a glicemia, positiva e muito forte com a idade, e, negativa e forte com a TFG. Não foi identificada correlação do SAGE com a PASp. Com base nas análises de sensibilidade e especificidade, o escore sete foi definido como o ponto de corte recomendado para indicar a realização da análise da rigidez arterial considerando como diagnóstico positivo a VOP ≥ 10 m/s. Já para VOP ≥ 9,08 m/s e ≥ 7,30 m/s, os pontos de corte foram, respectivamente, 5 e 2.

No presente estudo, o fato de os pacientes serem não hipertensos, o qual é um dos parâmetros que compõem o escore SAGE, não resultou em um ponto de corte menor na análise incluindo a VOP ≥ 10 m/s, assim como no estudo de desenvolvimento do escore.^5^ O ponto de corte do SAGE foi semelhante ao definido pelo estudo original realizado com pacientes hipertensos caucasianos de origem europeia,^5^ e ao estudo feito com 837 brasileiros hipertensos,^7^ que obtiveram o ponto de corte de oito. Ainda, o ponto de corte foi igual ao do estudo feito com 1816 japoneses hipertensos,^6^ em que o ponto de corte foi sete. Isso pode ser justificado pelo fato de que, mesmo não apresentando hipertensão arterial, que é um dos fatores que contribuem com a pontuação do SAGE, todos os participantes com VOP ≥ 10 m/s apresentavam idade elevada (≥ 70 anos), o que já atribui seis pontos ao escore. A relação entre o aumento da idade cronológica e a rigidez arterial já está bem estabelecida na literatura,^17 , 18^ uma vez que, concomitantemente ao envelhecimento cronológico do organismo, ocorre também o envelhecimento vascular, culminando no aumento da rigidez arterial.^18 - 24^ Por outro lado, é possível que o estabelecimento de pontos de corte para estratos de PAS menores que 140 mmHg, e a inclusão de outros parâmetros no escore, como o colesterol, possa otimizar ainda mais a aplicabilidade desse escore para a população de normotensos e pré-hipertensos.

Além do fator idade, verificou-se que a maioria dos indivíduos com VOP ≥ 10 m/s apresentou dislipidemia. Esse fator de risco, apesar de não ser contemplado pelo escore SAGE, também contribui para o desenvolvimento e a progressão da rigidez arterial. As taxas basais triglicérides (TG) e a razão entre os níveis TG e de colesterol de alta densidade (TG/HDL) apresentaram associação independente com o aumento persistente da VOP e com a incidência de VOP elevada em homens saudáveis acompanhados por 4,1 anos.^25^

Analisamos também os percentis 75 e 50 da VOP considerando que a VOP ≥ 10 m/s já identifica lesão de órgão-alvo,^9 , 15^ e nessa população não hipertensa, valores da VOP menores que 10 m/s já representam um aumento da rigidez arterial e por consequência, do risco cardiovascular.^26^ Foram identificados 25 e 51 participantes com VOP acima dos percentis 75 e 50, respectivamente.

Outro aspecto a se considerar é a aplicação do escore SAGE com um ponto de corte mais baixo, por exemplo cinco, como estratégia para identificar valores de VOP maiores que o percentil 75, o que resultaria na identificação de um a cada quatro não hipertensos. Já o uso do escore dois, definido para o percentil 50, não seria tão viável, pois recomendaria quase todos os pacientes para a realização do exame de análise da rigidez arterial.

Ao nosso ver, a avaliação do risco de aumento na rigidez arterial mesmo em indivíduos não hipertensos, representa uma enorme janela de oportunidade para identificar precocemente lesões subclínicas e possibilitar o estabelecimento de estratégias não farmacológicas e/ou farmacológicas com o objetivo de otimizar a prevenção e proteção cardiovascular.

Na investigação do papel dos biomarcadores na prevenção primária, a avaliação da rigidez arterial foi recomendada também para pacientes com diabetes, dislipidemias e com doença renal crônica, reforçando a influência desses outros fatores de risco na VOP.^1^ A taxa de glicemia e a TFG foram identificadas como preditores independente da VOP,^5^ e no presente estudo elas apresentaram correlação com o SAGE. Ainda, a redução da complacência e/ou da distensibilidade dos vasos ocorre independentemente da pressão arterial na presença de outros fatores de risco, dentre eles, a diabetes mellitus, o envelhecimento cronológico, a síndrome metabólica, a obesidade, a doença arterial periférica e a doença renal em estágio final.^27^

Além disso, apesar de a maioria dos estudos apresentarem a hipertensão como um dos principais fatores de risco para o aumento da rigidez arterial, o próprio aumento da rigidez prediz a ocorrência de hipertensão arterial e contribui para sua patogênese, reforçando a importância de analisar a VOP mesmo em indivíduos não hipertensos.^17 , 18 , 28 - 34^ Em um estudo de seguimento de uma coorte de Framingham com 1048 participantes, acompanhados por quatro a 10 anos, a VOP, avaliada pelo método tonométrico carotídeo-femoral, foi identificada como preditora de hipertensão arterial, enquanto o aumento da pressão arterial não foi preditor da rigidez elevada. E cada aumento de um desvio-padrão na VOP carotídeo-femoral, aumenta em 30% o risco de desenvolver a hipertensão arterial.^27^

Uma das limitações do estudo é que o escore SAGE foi desenvolvido para indivíduos hipertensos e, por isso, não há pontuação diferenciada para normotensos e pré-hipertensos. Por outro lado, cria-se aqui a oportunidade do desenvolvimento de escores específicos para essa população, por exemplo com atribuição de uma pontuação também para os indivíduos pré-hipertensos, visto que eles já apresentam um risco aumentado para as doenças cardiovasculares. ^35 - 37^ Outra limitação refere-se à idade dos participantes em nossa amostra, principal fator correlacionado à chance de VOP aumentada nesse modelo. Portanto, seria importante a realização de novos estudos incluindo amostras maiores e com mais indivíduos em cada faixa etária para verificar se, em indivíduos não hipertensos, seria mais recomendado medir a VOP apenas pelo critério da idade (≥ 70 anos) em vez do cálculo do escore.

## Conclusões

Na amostra estudada, a aplicação do escore SAGE foi capaz de identificar indivíduos com maior chance de apresentar rigidez arterial para diferentes pontos de corte de VOP. Entretanto, o desenvolvimento de um escore específico para a população de normotensos ou pré-hipertensos se faz necessário, e pode contribuir de maneira significativa na incorporação da análise de risco de envelhecimento vascular nessa população.
