# Supplementary material for: Escore SAGE em Normotensos e Pré-Hipertensos: Uma Prova de Conceito
Source: Arq Bras Cardiol. 2023 Feb 13;120(2):e20200291. [Article in Portuguese] doi: 10.36660/abc.20220291 (PMC9972660; doi:10.36660/abc.20220291)
Supplement: Translation

# Translation: SAGE Score in Normotensive and Pre-Hypertensive Patients: A Proof of Concept

**Authors:** Rayne Ramos Fagundes Rigonatto, Priscila Valverde Oliveira Vitorino, Adriana Camargo Oliveira, Ana Luiza Lima Sousa, Paulo César Brandão Veiga Jardim, Pedro Miguel Guimarães Marques Cunha, Eduardo Costa Duarte Barbosa, Panagiotis Xaplanteris, Charalambos Vlachopoulos, Weimar Kunz Sebba Barroso

**Affiliations:** 1 Brasil Universidade Federal de Goiás – Programa de Pós-graduação em Ciências da Saúde, Goiânia, GO – Brasil; 2 Brasil Pontifícia Universidade Católica de Goiás – Escola de Ciências Sociais e da Saúde, Goiânia, GO – Brasil; 3 Brasil Universidade Federal de Goiás – Liga de Hipertensão Arterial, Goiânia, GO – Brasil; 4 Braga Portugal Escola de Medicina da Universidade do Minho, Braga – Portugal; 5 Brasil Complexo Hospitalar Santa Casa de Misericórdia de Porto Alegre, Porto Alegre, RS – Brasil; 6 Bélgica Université Libre de Bruxelles – Cardiology Department, Bruxelas – Bélgica; 7 Grécia National and Kapodistrian University of Athens School of Medicine – First University Department of Cardiology, Atenas – Grécia

**Keywords:** Hypertension, Biomarkers, Vascular Stiffness, Pulse Wave Analysis, Risk Factors

## Abstract

**Background:**

The SAGE score was developed to detect individuals at risk for increased pulse wave velocity (PWV). So far, studies have been focused on hypertensive patients.

**Objective:**

To assess the ability of the score to detect non-hypertensive and pre-hypertensive patients at risk for increased PWV.

**Methods:**

Retrospective cross-sectional study of analysis of central blood pressure data and calculation of the SAGE score of non-hypertensive and pre-hypertensive patients. Each score point was analyzed for sensitivity, specificity, positive and negative predictive values, using the cut-off point for positive diagnosis a PVW ≥ 10m/s, ≥9.08 m/s (75^th^percentile) and ≥7.30 m/s (50^th^percentile). A p<0.05 was considered statistically significant.

**Results:**

The sample was composed of 100 normotensive and pre-hypertensive individuals, with mean age of 52.64 ± 14.94 years and median PWV of 7.30 m/s (6.03 – 9.08). The SAGE score was correlated with age (r=0.938, p<0.001), glycemia (r=0.366, p<0.001) and glomerular filtration rate (r=-0.658, p<0.001). The area under the ROC curve was 0.968 (p<0.001) for PWV ≥ 10 m/s, 0.977 (p<0.001) for PWV ≥ 9.08 m/s and 0.967 (p<0.001) for PWV ≥ 7.30 m/s. The score 7 showed a specificity of 95.40% and sensitivity of 100% for PWV≥10 m/s. The cut-off point would be of five for a PWV≥9.08 m/s (sensitivity =96.00%, specificity = 94.70%), and two for a PWV ≥ 7.30 m/s.

**Conclusion:**

The SAGE score could identify individuals at higher risk of arterial stiffness, using different PWV cutoff points. However, the development of a specific score for normotensive and pre-hypertensive subjects is needed.

## Introduction

Pulse wave velocity (PWV) is a well-established biomarker in cardiovascular risk stratification and identification of subclinical lesions, and it can also be an indicator of target-organ lesion when higher than 10m/s.^1 - 4^However, PWV is still underutilized in clinical practice due to its high cost and low availability of the equipment.^5^

The SAGE score was developed to spread knowledge and the concept about the assessment of vascular aging and damage, based on four simple parameters – age, systolic blood pressure (SBP), fasting glycemia and glomerular filtration rate (GFR) – to calculate the probability of an individual developing increased arterial stiffness. Based on the values obtained, patients can be more assertively referred to central blood pressure (CBP) measurements and analysis of PWV.^5^

In the study in which the score was developed, the SAGE cut-off was calculated using the carotid-femoral PWV obtained from a hypertensive population.^5^Subsequently, the score was calculated using the brachial-ankle PWV in Japanese subjects with hypertension,^6^and more recently, it was calculated in Brazilian hypertensive individuals using the oscillometric method, which is a more commonly used method in Brazil.^7^

In 2021, a study^8^with 760 Chinese individuals developed a new clinical score using age, peripheral systolic blood pressure (pSBP), peripheral diastolic blood pressure (pDBP), weight and height, also aiming at identifying individuals with increased arterial stiffness. However, the study was conducted specifically on diabetic patients, and using brachial-ankle PWV measurements only.

There is still a gap in the literature regarding the use of these scores to identify increased arterial stiffness in non-hypertensive individuals that may already have increased PWV and increased risk of cardiovascular outcomes, and studies involving the oscillometric method, which is a low-cost, easy-to-use method.

Therefore, the aim of this study was to assess the ability of the SAGE score to identify individuals at high risk for increased PWV in a sample of normotensive and pre-hypertensive Brazilian individuals, as a proof of concept.

## Methods

### Study design and place

This was a cross sectional study in which medical records of patients attending two referral centers for diagnosis and treatment of hypertension in Brazil were analyzed.

### Population and sample

From September 2012 to November 2019, a total of 1594 measurements of CBP were made by the oscillometric method. Of these, we excluded:

- Measurements of patients younger than 18 years old;
- Measurements of patients with diagnosis of hypertension, defined as pSBP ≥ 140 mmHg and pDBP ≥ 90 mmHg, both obtained from CPB; or mean SBP ≥ 130 mmHg and/or mean DBP ≥ 80 mmHg PAS by ambulatory blood pressure monitoring (ABPM),^9 , 10^or use of anti-hypertensive drugs;
- Measurements of patients who did not have all clinical and laboratory data available for calculation of the SAGE score (pSBP, age, fasting glycemia, creatinine [for creatinine clearance according to the CKD-EPI)].^5^Laboratory tests should have been made from three months before to three months after the CBP measurement;
- Measurements of patients of creatinine clearance (calculated according to the CKD-EPI group) lower than 15mL/min/1.73m^2^.^5^

Then, the sample was composed of 100 normotensive or pre-hypertensive individuals, who had all data required for calculation of the SAGE score available ( Figure 1 ).

**Figure 1:** – Flowchart of patient selection. Source: Author, 2022.

### Study procedure

Electronic files of CBP measurements performed between September 2012 and November 2019 were identified. Then, medical files of these patients were analyzed for eligibility for the study.

Among the eligible patients, the following data were collected from the electronic files – date of birth, date of the CBP measurement, weight, height, peripheral and central SBP and DBP, peripheral and central pulse pressure, Augmentation Index (AIx) and PWV. For central and peripheral parameters, the mean of three measurements was considered for analysis.

In addition, the following data were collected: sex, smoking status, sedentary lifestyle, marital status, medications used, clinical diagnoses, and fasting glucose and creatinine levels obtained within three months before or after the CBP measurement. When glucose and creatinine levels were measured more than once within this period, those obtained on the closest date to the CBP measured were used for analysis.

Body mass index was calculated using the formula: $\text{weight}(Kg)/[\mathrm{height}(m){]}^{2}$ .^11^

### Assessment of central blood pressure

Measurements of CBP were performed using the Mobil-O-Graph^®^(IEM, Stolber, Germany) and the Dyna MAPA AOP^®^(Cardios, São Paulo, Brazil). This evaluation is performed non-invasively; pSBP and DBP are measured using a sphygmomanometer and the ARCSolver algorithm is used to derive central pressure.^12^

The assessment of PWV using the sphygmomanometer and the oscillometric method yields comparable values to those obtained invasively using the intra-aortic catheter,^12^in addition to being more reproducible than the devices used for assessment of carotid-femoral PWV.^13^The method has also been validated for assessment of central SBP compared with the assessment by the invasive method and the tonometric method.^14^An increase in arterial stiffness compatible with target organ damage was detected by PWV ≥ 10m/s.^9 , 15^

### Calculation of the SAGE score

The SAGE score is defined based on four variables – fasting glucose, pSBP, age, and estimated GFR ( Figure 2 ).

**Figure 2:** – SAGE score classification and its four variables; translated from Xaplanteris et al.5

For example, an individual with a SBP of 145 mmHg, glycemia of 110 mmHg, 65 years old and GFR of 69 mL/min/1.73m^2^will be assigned a SAGE score of eight and, therefore, as defined by the study in which the score was developed, will be referred for assessment of increased arterial stiffness due to higher risk of its occurrence.^5^

The SAGE score was calculated for each participant. Measurements of pSBP were obtained from the CBP measurement; age was calculated by the difference between the date of birth and the date that of the CBP measurement. Glycemia was obtained from patient medical record and GFR was calculated using the CKD-EPI creatinine equation (2021), using serum creatinine levels obtained from the medical record.

### Statistical analysis

Data were collected by two investigators using a form developed with the Epidata software version 3.1.^16^The program was also used for validation of the form in terms of potential inconsistencies and errors.

Data analysis was made using the Statistical Package for Social Science (SPSS) version 23.0. Normality of data distribution was tested using the Kolmogorov-Smirnov test; descriptive analysis was performed using mean and standard deviation and using median and interquartile range for parametric and non-parametric data, respectively. Qualitative data were described as absolute and relative frequencies.

The correlation between SAGE score and the four variables of the score was assessed by the Spearman correlation.

Analysis of sensitivity, specificity, positive predictive value and negative predictive value was made for each SAGE rating. A positive diagnosis was defined as the presence of three PWV measurements ≥ 10m/s and ≥ 50^th^and 75^th^percentiles, which correspond to 7.3 and 9.8 m/s, respectively. For each of these values, a ROC curve was constructed to define the best cut-off point for the SAGE score, *i.e* ., the one with the highest sensitivity and specificity to detect patients at higher risk for increased PWV. A p<0.05 was considered statistically significant.

### Ethical aspects

The study was conducted according to the resolution number 466/12 and approved by the ethics committee of the General Hospital of the Federal University of Goias (approval and amendment number 1.500.463 and 3.792.750, including approval of the waiver of the consent form).

## Results

Data of 100 participants were analyzed, with mean age of 52.64 ± 14.94 years. Most patients were male, and had dyslipidemia, with optmial blood pressure and PWV lower than 8m/s ( Table 1 ).

**Table 1:** – Clinical and sociodemographic characteristics of participants (n=100)

Variable	n/%
**Sex**	
Female	45
Male	55
**Marital status**	
Living without a partner	29
Living with a partner	57
Not reported	14
**Age**	
< 50 years	42
50 - 59 years	24
60 - 69 years	18
≥70 years	16
**Risk factors**	
Current smoking	5 / 5.3%*
BMI > 30Kg/m^2^	24
Diabetes mellitus	11 / 11.7%*
Dyslipidemia	53 / 65.4%*
**Total cholesterol**	
< 150 mg/dl	29
150 - 199 mg/dl	41
200 - 249 mg/dl	18
250 - 299 mg/dl	4
≥ 300 mg/dl	1
Not reported	7
**LDL**	
≤50 mg/dl	8
51–69 mg/dl	10
70–99 mg/dl	26
100–129 mg/dl	31
≥130 mg/dl	16
Not reported	9
**Triglycerides**	
<150 mg/dl	63
≥150 mg/dl	26
Not reported	11
**Glycemia**	
< 126 mg/dl	94
≥ 126 mg/ dl	6
**Glomerular filtration rate**	
30 - 59	5
60 - 89	48
≥ 90	47
**Classification of blood pressure**	
Optimal BP	46
Normal BP	34
Pre-hypertension	20
**Arterial stiffness**	
PWV < 8 m/s	59
PWV 8 - 10 m/s	29
PWV > 10 m/s	12
**Central pressure parameters**	**Média (DP) / Mediana (25 – 75)**
pSBP (mmHg)	119.43 (9.59)
pDBP (mmHg)	75.50 (67.00 – 79.75)
pPP (mmHg)	45.00 (39.00 – 52.00)
cSBP (mmHg)	109.15 (9.38)
cDBP (mmHg)	77.00 (67.25 – 81.00)
cPP (mmHg)	32.00 (29.00 – 39.00)
AI (%)	18.87 (11.30)
PWV (m/s)	7.30 (6.03 – 9.08)

*These data were not available from some of the patients and the frequency was then different from the number; AI (%): augmentation index; HDL: high-density lipoprotein; BMI: body mass index; LDL: low-density lipoprotein; cDBP: central diastolic blood pressure; pDBP: peripheral diastolic blood pressure; cSBP: central systolic blood pressure; pSBP: peripheral systolic blood pressure; cPP: central pulse pressure; pPP: peripheral pulse pressure; PWV: pulse wave velocity

The most frequent SAGE score in the sample was 0, followed by 1 and 2 ( Figure 3 ). Patients’ characteristics that could justify the scores were analyzed; of 13 participants, 12 were aged 70 years or older and had fasting glucose < 126 mg/dL and GFR of 60 - 89 mL/min/1.73m^2^. The other patient was aged between 60 and 69, had fasting glucose ≥ 126mg/dL and the same GFR.

**Figure 3:** – Relative frequency of the SAGE scores of the study patients (n=100).

Among patients with arterial stiffness (PWV ≥ 10 m/s), the most frequent score was seven ( Figure 4 ). All patients with PWV ≥ 10 m/s (n=13, 100%) were aged 70 years or older, and had fasting glucose < 126 mg/dL; 10 (76.9%) had GFR between 60 and 89 mL/min/1.73m^2^, and three (23%) between 30 e 59 ml/min/1,73m^2^. Eleven (84,6%) had dyslipidemia.

**Figure 4:** – Absolute and relative frequency of SAGE score ratings of the study patients; (A) patients with pulse wave velocity (PWV) ≥ 10 m/s, (B) patients with PWV ≥ 9.08 m/s and (C) patients with PWV ≥ 7.30 m/s.

Analysis of the 75^th^percentile (9.08 m/s) and the 50^th^percentile of the PWV (7.3 m/s), the most frequent SAGE score was also seven. Among patients with PWV ≥ 9.08 m/s, 88% had fasting glucose < 126 mg/dL, 64% were aged 70 years or older (and the others between 60 and 69 years), and 80% had GFR between 60 and 89 mL/min/1.73m^2^.

Distribution of SAGE parameters by age, pSBP, fasting glucose, and GFR (according to the CKD-EPI group) ( Figure 5 ) showed a positive correlation with age and glucose levels, and a negative correlation with GFR. No correlation was found between SAGE and pSBP.

**Figure 5:** – Distribution of SAGE score by age (A) peripheral systolic blood pressure (B), glycemia (C) and glomerular filtration rate (D).

In the analysis of the ROC curve, the area under the curve for PWV ≥ 10 m/s was 0.968 (p<0.001), for PWV ≥ 9.08 m/s was 0.977 (p<0.001) and for PWV ≥ 7.30 m/s was 0.967 (p<0.001) ( Figure 6 ).

**Figure 6:** – ROC curve of the SAGE score for pulse wave velocity (PWV) ≥ 10 m/s (A), PWV ≥ 9.08 m/s (B) and PWV ≥ 7.30 m/s.

According to the sensitivity and specificity analysis ( Table 2 ), for individuals with arterial stiffness (PWV ≥ 10 m/s), a SAGE score of seven showed high specificity (95.40%) associated with a sensitivity of 100% and a negative predictive value of 100%. Considering the percentile 75^th^(PWV ≥ 9.08 m/s), the cut-off point for SAGE would be ≥ 5, with a sensitivity of 96% and specificity of 94.7%. For the median PWV (≥ 7.30 m/s), the cut-off point would be lower (SAGE score of two).

**Table 2:** – Sensitivity and specificity of the SAGE score by cutoff point and pulse wave velocity value

	SAGE	Sensitivity	Specificity	PPV/ Correctly classified	NPV
**PWV ≥ 10 m/s**	0	100.00%	0.00%	13.00%	-
	1	100.00%	27.59%	17.11%	100.00%
	2	100.00%	47.13%	22.03%	100.00%
	3	100.00%	63.22%	28.89%	100.00%
	4	100.00%	73.56%	36.11%	100.00%
	5	100.00%	82.76%	46.43%	100.00%
	6	100.00%	91.95%	65.00%	100.00%
	7	100.00%	95.40%	76.47%	100.00%
	8	23.08%	98.85%	75.00%	89.60%
	9	0.00%	98.85%	0.00%	86.90%
**PWV ≥ 9.08 m/s**	0	100.00%	0.00%	25.00%	-
	1	100.00%	32.00%	32.89%	100.00%
	2	100.00%	54.70%	42.40%	100.00%
	3	100.00%	73.30%	55.60%	100.00%
	4	100.00%	85.30%	69.40%	100.00%
	5	96.00%	94.70%	85.70%	98.60%
	6	72.00%	97.30%	90.00%	91.30%
	7	68.00%	100.00%	100.00%	90.40%
	8	16.00%	100.00%	100.00%	78.10%
	9	4.00%	100.00%	100.00%	75.80%
**PWV ≥ 7.30 m/s**	0	100.00%	0.00%	51.00%	-
	1	100.00%	48.98%	67.11%	100.00%
	2	98.00%	81.60%	84.70%	97.60%
	3	82.40%	93.90%	93.30%	83.60%
	4	68.60%	98.00%	97.20%	75.00%
	5	54.90%	100.00%	100.00%	68.10%
	6	39.20%	100.00%	100.00%	61.30%
	7	33.30%	100.00%	100.00%	59.00%
	8	7.80%	100.00%	100.00%	51.00%
	9	2.00%	100.00%	100.00%	49.50%

*NPV: negative predictive value, PPV: positive predictive value; PWV: pulse wave velocity.*

## Discussion

In the present study, we found that a SAGE score of zero was the most frequent in this sample of non-hypertensive patients. On the other hand, a SAGE score of seven was the most common among patients with PWV ≥ of 7,3 m/s, 9,08 m/s or 10 m/s. The SAGE score showed a moderate positive correlation with glycemia, a very strong positive correlation with age, and a strong negative correlation with GFR. No correlation was observed between SAGE and pSBP. Based on the analysis of sensitivity and specificity, the score seven was defined as arterial stiffness considering a PWV ≥ 10 m/s as the positive diagnosis. For PWV ≥ 9.08 m/s and ≥ 7.30 m/s, the cut-off points were 5 and 2, respectively.

In the present study, the fact that the patients were not hypertensive, which is one of the parameters of the SAGE score, did not lead to a lower cut-off point in the analysis including PWV values ≥ 10 m/s, like the study in which the score was developed.^5^The cut-off point in our study was similar to that established in the original study with European Caucasian hypertensive patients^5^and to the Brazilian study with 837 hypertensive patients^7^which established a cut-off of eight. In addition, it was equal to that reported in a Japanese study with 1,816 hypertensive individuals,^6^in which the cut-off was seven. This may be justified by the fact that, even though these patients do not have hypertension, which is a condition that already contributes to the SAGE score, all patients with PWV ≥ 10m/s were aged 70 years or older, which already contributes to six points to the score. The relationship between aging arterial stiffness is already well established in the literature^17 , 18^since concomitantly with chronological aging, vascular aging occurs, culminating in increased arterial stiffness.^18 - 24^On the other hand, one may consider that the establishment of cutoffs for blood pressure levels lower than 140 mmHg and the inclusion of other parameters, like cholesterol, may optimize the applicability of this score in normotensive and pre-hypertensive populations.

In addition to the age factor, most individuals with PWV ≥ 10 m/s had dyslipidemia. Although this risk factor was included in the SAGE score, it also contributes to the development of arterial stiffness. Baseline triglyceride (TG) levels and the TG/high-density lipoprotein (HDL) ratio are independently associated with the persistent increase in PWV and the incidence of increased PWV in healthy men followed-up for 4.1 years.^25^

We also analyzed the 75^th^and the 50^th^percentiles, since PWV ≥ 10m/s can already be an indicator of target-organ damage.^9 , 15^Also, in this population of non-hypertensive individuals, PWV values lower than 10m/s already represents an increase in arterial stiffness and consequently in cardiovascular risk.^26^Twenty-five and 51 participants were found to have PWV above the 75^th^and 50^th^percentiles, respectively.

Another aspect to be considered is the application of the SAGE score with a lower cut-off point (e.g. five) as a strategy to detect PWV values above the 75^th^percentile, which would result in the identification of one in every four non-hypertensive individuals. The use of a score of two, defined for the 50^th^percentile, would not be feasible, as almost all patients would have to be referred to assessment of arterial stiffness.

In our opinion, the risk assessment of increased arterial stiffness even in non-hypertensive individuals represents a great window of opportunity to identify early subclinical lesions and to establish non-pharmacological and pharmacological strategies aiming at optimizing cardiovascular protection and prevention.

In the investigation of the role of biomarkers in primary prevention, the assessment of arterial stiffness was also recommended for patients with diabetes, dyslipidemias and chronic renal disease, reinforcing the influence of these risk factors on PWV.^1^Both glycemia and GFR were identified as independent predictors of PWV and,^5^in this present study, they were correlated with SAGE. Also, the reduction in arterial compliance and/or distensibility occurs independently of blood pressure in the presence of other risk factors, including diabetes mellitus, chronological aging, metabolic syndrome, obesity, peripheral artery disease, and end-stage renal disease.^27^

Besides, although most studies have reported that hypertension is one of the main risk factors for increased arterial stiffness, this increase, in turn, is a predictor of hypertension and contributes to its pathogenesis, reinforcing the importance of assessing the PWV even in non-hypertensive individuals.^17 , 18 , 28 - 34^In a follow-up study of the Framingham cohort with 1048 participants, followed-up for four to 10 years, carotid-femoral PWV, obtained by tonometry, was identified as a predictor of arterial hypertension, while the increase in blood pressure was not a predictor of increased arterial stiffness. A one standard deviation in carotid-femoral PWV increased by 30% the risk of arterial hypertension.^27^

One limitation of this study is that the SAGE score was developed for hypertensive subjects, and, for this reason, there are no differential ratings between normotensive and pre-hypertensive patients. On the other hand, an opportunity for the development of specific scores for this population is warranted, including the assignment of ratings to pre-hypertensive patients also, considering their increased risk for cardiovascular diseases.^35 - 37^Another limitation was the age of the study population, which is the main factor correlated to increased PWV in this model. Thus, further studies are needed including larger samples of individuals in different age ranges to determine whether it would be more appropriate to measure PWV of non-hypertensive patients using the criterion of age (≥70 years) rather than the score calculation.

## Conclusions

In the study sample, the SAGE score could identify patients at higher risk of arterial stiffness by different PWV cutoffs. However, the development of a specific score for non-hypertensive and pre-hypertensive patients is needed and could contribute significantly to the implementation of the analysis of the risk of vascular aging in this population.
